# A Reflection Upon the Contribution of Retinal and Cortical Electrophysiology to Time of Information Processing in Psychiatric Disorders

**DOI:** 10.3389/fpsyt.2022.856498

**Published:** 2022-04-05

**Authors:** Thomas Schwitzer, Marion Leboyer, Raymund Schwan

**Affiliations:** ^1^Pôle Hospitalo-Universitaire de Psychiatrie d'Adultes et d'Addictologie du Grand Nancy, Centre Psychothérapique de Nancy, Laxou, France; ^2^INSERM U1254, IADI, Université de Lorraine, Nancy, France; ^3^Faculté de Médecine, Université de Lorraine, Vandœuvre-lès-Nancy, France; ^4^Fondation FondaMental, Créteil, France; ^5^Université Paris Est Creteil (UPEC), AP-HP, Hôpitaux Universitaires ≪ H. Mondor ≫, DMU IMPACT, FHU ADAPT, INSERMU955, IMRB, Translational Neuropsychiatry Laboratory, Creteil, France

**Keywords:** electrophysiology, retina, electroretinogram, precision medicine, electroencephalography

## Introduction

Recent advances in psychiatric research support the use of retinal electrophysiology as a relevant approach to indirectly study neural functioning and to identify relevant markers of diagnosis, prognosis, treatment response, detection of high-risk subjects and identification of subgroups of patients in psychiatric disorders (1–9). Using retinal electrophysiological techniques named electroretinogram (ERG), retinal dysfunctions were observed in patients with psychiatric disorders and substance uses (1, 5, 7). Several abnormalities detected with ERG concern the retinal time, i.e., the time necessary for the firing of retinal neurons also called implicit time or peak time (1, 5, 7). These results suggest modifications of information processing speed at the retinal level. Since retinal neurons share similar functional properties with brain neurons, this inform on information processing speed in the brain. Based on these results, the question arises as to whether these anomalies of retinal times are also detected in the brain or are corrected throughout the visual pathways. However, very few studies have investigated simultaneously brain electrical activity of visual cortex and retinal electrical activity with electrophysiological techniques. Here, we argue for the use of combined and synchronized retinal and cortical electrophysiology by ERG and visual evoked potentials (VEP). To this end, we present evidences based on anatomy, physiology and methodology. From these measures, we suggest that the retino-cortical time (RCT), which is an integrated parameter based on both retinal and cortical time, may be of particular interest in psychiatry although it is currently not used (10). Finally, we support the use of signal processing and machine learning tools applied on combined retinal and cortical measures for precision medicine in psychiatry.

## Anatomical and Physiological Evidences

The retina is an anatomical and developmental extension of the central nervous system (SNC) and it is organized in layers of specialized neurons (11). Retinal neurons display similarities to brain neurons in terms of anatomy, functioning, central damages and response to insult (11). Retinal neurons are sensitive to damages such as neurotransmission dysfunctions, neurodegeneration, inflammation and autoimmunity, similar to brain neurons. Retinal damages reflect the condition of the brain. CNS disorders have also manifestations in the retina that reflect brain pathological conditions. Thus, the study of retinal neurons functioning can help the diagnosis and the understanding of brain pathophysiological conditions in psychiatric disorders (4, 6). Retinal and cortical neurons are interconnected by the optic nerve which is constituted by the axons of the retinal ganglion cells (11). Visual processing begins in the retina with the absorption of light by the photopigments of the photoreceptors -rods and cones-, thus initiating the conversion of light into neural activity, phenomenon called phototransduction (11). The electrical signal is then transmitted throughout the retinal pathways by successive firings of retinal neurons such as photoreceptors, bipolar cells and ganglion cells (11). Then, the electrical signal is relayed in the form of action potentials to the brain by the optic nerve–axons of the ganglion cells- and transmitted to the visual occipital cortex where firings of cortical neurons occur to elicited visual evoked potentials (VEP). The cortical time -the response time of visual cortex- can be derived from VEP (11). Thus, firing time of both retinal and cortical neurons inform on the well physiological functioning throughout the visual pathways. Retinal and cortical time allow to study the speed of visual information processing from the retina to the brain, to detect acceleration or slowdown of visual processing and to localize potential anomalies between retinal and cortical stage. The RCT is the difference between the cortical and retinal time (10). Previous works support its relevance for the study of optic pathways functioning, which can be altered in psychiatric disorders (10). The visual function and most specifically low-level vision are good candidates for conceptualizing the neural impact of psychiatric disorders and substance uses (12–14). Thus, the time of information processing throughout the visual pathways and recorded with combined and synchronized ERG and VEP recordings may give good indicators of alterations in the speed of information processing in the brain, which are frequently observed in psychiatric disorders and substance uses (15–17).

## Methodological Evidences

The functioning of retinal neurons can be assessed by ERG whereas the functioning of cortical neurons can be assessed by electroencephalography (EEG) (18–23). VEP represent the electrical activity evoked by cortical neurons of visual pathways following visual stimulations (20). Interestingly, ERG and VEP share similar as well as complementary characteristics favoring their combined usage (23). They are rapid and non-invasive techniques recording the electrical bio-potential evoked by retinal and cortical neurons in response to various types of visual stimulations such as flashes or checkerboards stimulations (18, 20, 21, 23). Typical traces obtained with ERG and VEP recordings share similar morphology. From these typical traces, waves of interest can be derived and from these waves, main parameters can be extracted namely amplitude (μvolt) and implicit time –also called peak time, response time or latency- (ms) (18, 23). Amplitude and implicit time are the result of different cellular mechanisms. Amplitude is linked to the number of cells involved in the visual response and represents its quantitative properties whereas the implicit time is associated with the qualitative properties of neurons involved in the visual response. Implicit time is a robust parameter extracted from ERG and VEP since it is little influenced by acquisition and instrumentation techniques as well as by inter or intra individual variations in non-pathological conditions. As a consequence, it is highly reproducible between subjects and independent of recording conditions. Interestingly, ERG and EEG can be measured simultaneously and EEG recordings can be synchronized with ERG recordings, which facilitate the measure of both retinal and cortical parameters and also allow the measure of the RCT. This enables the study of the functional properties and the time of information processing throughout the visual pathways.

## Retinal Dysfunctions in Psychiatric Disorders and Substance Uses

Retinal dysfunctions are detected with ERG in several psychiatric disorders -major depressive disorders, bipolar disorders, schizophrenia, autism spectrum disorder-, to name a few- and substance uses -cannabis, tobacco, alcohol, cocaine use-, for example (1–5, 7). Interestingly, several of these alterations concern the retinal time, i.e., implicit time, arguing for acceleration or slowdown of retinal processing in these disorders or substance uses (1). For example, in regular cannabis users, delayed retinal processing was observed at the ganglion and bipolar cells levels, as showed by increased pattern ERG N95 implicit time and by increased b- and d-wave implicit time of the flash ERG (3, 24–28). In tobacco users, an increase in b-wave implicit time of the flash ERG was observed as well as an increases in P1 and N2 implicit time of the multifocal ERG (mfERG) (29). Similarly, the P1 implicit time of ring 1 on mfERG was reduced after alcohol administration (30). In major depressive disorders, a delayed signaling in the central retina and an hyperreactivity to light in the periphery were observed as showed by an increase in pattern ERG P50 implicit time and a decrease in a- and b-wave implicit time of the flash ERG in dark and light adapted conditions (31). In schizophrenia, increase in PERG P50 and N95 implicit time and modulations of a- or b-wave implicit time in scotopic and photopic conditions were observed (32–34). These anomalies observed at the retinal level may inform on pathophysiological mechanisms of neural functioning involved in psychiatric disorders and substance uses. Pathophysiology of these disorders can imply neurotransmission dysregulation, inflammation, neurodegeneration, auto-immunity, to name a few (35–42). Interestingly, these mechanisms can be associated with modifications in the speed of information processing in the brain. As the retina is a window to the brain, alterations of time of information processing of retinal neurons could give knowledge on the speed of information processing in brain neurons. Of interest, abnormalities observed in retinal time of information processing could give information on the time of information processing of high-level cognitive functions since retinal dysfunctions were correlated with cognitive dysfunctions (43, 44).

## Interest of Combined Retinal and Cortical Electrophysiology

**Figure 1:**
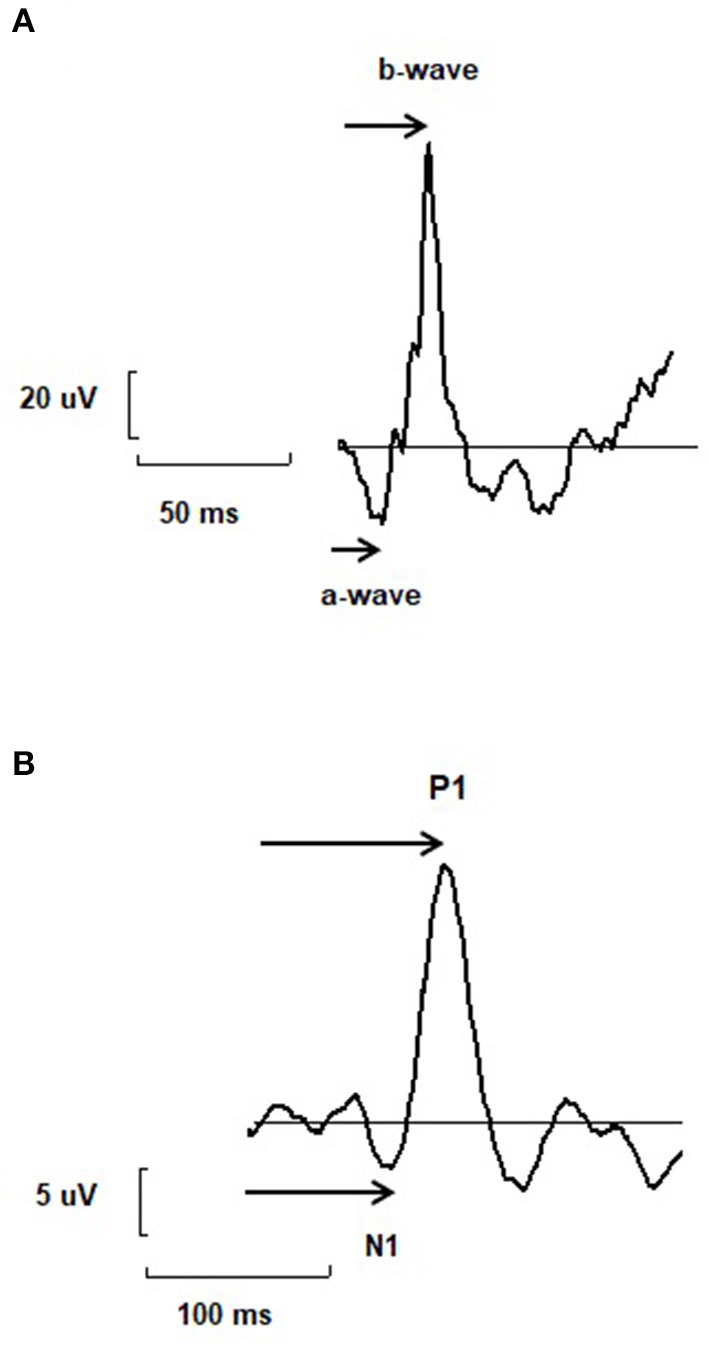
**(A)** A typical trace of electroretinogram (ERG) with two main components named a- and b-wave. The arrow represents the way the implicit time or peak time of each wave is measured. **(B)** A typical trace of electroencephalogram (EEG) recorded using visual stimulations and called visual evoked potential (VEP), with two main components N1 and P1. The arrow represents the way the implicit time or peak time of each wave is measured. The retino-cortical time is the difference between the implicit time of N1 and b-wave.

Since retinal information processing is impaired in psychiatric disorders and substance uses, the question arises as to whether these anomalies of retinal time are also detected in the brain or are corrected throughout the visual pathways. To this end, the study of combined ERG and VEP is of particular interest. However, despite of anomalies in time responses of retinal signaling, there are very few studies that have simultaneously evaluated the time response of both retinal and cortical activity by VEP and ERG. Combined and synchronized measures of retinal and cortical function by ERG and VEP may give additional information of pathophysiological mechanisms underlying psychiatric disorders, may allow to locate anomalies throughout the visual pathways and may eventually give biomarkers to produce biosignatures for precision medicine in psychiatry (45–47). In addition to the relevance of parameters derived from isolated ERG and VEP recordings such as retinal and cortical times, the measure of combined and synchronized ERG and VEP allows to evaluate the retino-cortical time. It may be particularly relevant and may give a novel indicator allowing the measure of the time of visual processing between the retina and the brain (10). The RCT is the time which elapses between the beginning of the electrical response of the retina –as measured by the b-wave of the flash ERG- and the onset of the electrical response of the cortical visual center –as measured by the first negative wave of the EEG typical trace, here called N1 (10) (Figure 1). It provides information on the conductivity of the visual pathways as well as on the reactivity of the cortical visual center (10). It represents a robust indicator since it is an integrated parameter derived from both retinal and cortical criterion. It allows the assessment of the functioning of the central visual system and is a marker of the optical path functioning. It was previously described as particularly crucial for optic path disorders (10). Interestingly, optic path disorders are observed in psychiatric disorders such as schizophrenia, bipolar disorders, major depressive disorders, autism spectrum disorders and substance uses (12, 13, 48–51). By giving additional and complementary information of the time of information processing in central neurons, the RCT may help to a better understanding of pathological mechanisms of brain neurons involved in psychiatric disorders. It may also provide a new electrophysiological marker, which can be added in sets of biomarkers with other retinal and cortical markers in order to produce biosignatures in psychiatric disorders. To the best of our knowledge, one study evaluated both retinal and cortical electrophysiology in psychiatric disorders (52). In this study, PERG and VEP of the occipital cortex with an Oz vs. FPz derivation were recorded with checkerboard stimuli in 40 MDD patients and 28 controls. MDD patients displayed reduced PERG and VEP amplitudes compared to control subjects. PERG and VEP amplitudes were correlated with psychometric measures for severity of depression. These results suggest that both retinal and cortical responses are altered in MDD.

## Discussion

In addition to their relevance in psychiatric disorders, retinal, cortical and retino-cortical time may be interesting in neuropsychiatric disorders and especially in cognitive impairments or dementia, which can be associated with the course of psychiatric disorders (17, 40, 53). In these cases, the time of information processing is often altered and may impact the global functioning and quality of life. To this end, retinal and cortical electrophysiology could offer relevant indicators of cognitive deficits. In order to produce robust indicators, signal processing and machine learning techniques are promising tools for precision psychiatry (46). All visual electrophysiological data can be analyzed with signal processing and machine learning techniques to produce biosignatures for a better identification of subgroups of patients. When applied on individuals and populations, they will provide better diagnosis, prognosis, treatment and detection of high-risk subjects of mental disorders (46). Interestingly, recent works have already focused on the use of signal processing and machine learning tools applied on visual electrophysiological data in psychiatric disorders and substance uses. For example, signal processing and machine learning tools applied on PERG data provided discrimination between MDD patients and controls at the inclusion and reflected the efficacy of the treatment at the end of the follow-up at week 12 after treatment (54). Similarly, machine learning algorithm and discriminant analysis of EEG proved to be useful in predicting the efficacy of antidepressants based on the main symptoms of depression and the characteristics of the pre-treatment EEG in MDD (55). Finally, signal processing based on Fourier transform was applied on retinal electrophysiological data and used to isolate the retinal background noise. The retinal noise was different between regular cannabis users with high and low alcohol use supporting identification of subgroups of cannabis users (56). Electrophysiology is already suggested as a technique related to precision psychiatry. Combined measures of ERG and VEP could be added as electrophysiological techniques in order to extract relevant biomarkers. To this end, visual electrophysiology should be evaluated in various pathological conditions, subgroups of patients and specific clinical situations that may require clinical decision support to determine its relevance (9). In future studies, visual electrophysiology should be coupled with other electrophysiological measures such as electrocardiography (ECG) and with neurophysiological measures to enhance the powerfulness of each measure. Finally, molecular mechanisms of pathology and reaction to pharmacological agents could be assessed in future studies in order to confirm pathophysiological conditions in the CNS observed with visual electrophysiology.

To conclude, future studies using electrophysiological techniques in psychiatric disorders and substance uses will include combined and synchronized measures of retinal and cortical electrophysiology by ERG and VEP as well as the RCT. Studying visual electrophysiology from the retina to the brain is promising for a better understanding of pathophysiological mechanisms underlying psychiatric disorders and could also provide additional electrophysiological markers for precision medicine in psychiatry.

## Author Contributions

TS wrote the manuscript and all authors listed have made a critical review to the work and approval it for publication.

## Funding

The open access publication fee was provided by the Centre Psychothérapique de Nancy.

## Conflict of Interest

The authors declare that the research was conducted in the absence of any commercial or financial relationships that could be construed as a potential conflict of interest.

## Publisher's Note

All claims expressed in this article are solely those of the authors and do not necessarily represent those of their affiliated organizations, or those of the publisher, the editors and the reviewers. Any product that may be evaluated in this article, or claim that may be made by its manufacturer, is not guaranteed or endorsed by the publisher.
